# Editorial: Novel modalities in cancer diagnostics and therapeutics

**DOI:** 10.3389/fphar.2022.1101506

**Published:** 2022-11-30

**Authors:** Serena Li Zhao, Dong-Hua Yang, Haichang Li

**Affiliations:** ^1^ Department of Medicinal Chemistry and Pharmacognosy, The Ohio State University College of Pharmacy, Columbus, OH, United States; ^2^ Department of Pharmaceutical Sciences, College of Pharmacy and Health Sciences, St. John’s University, New York, NY, United States; ^3^ Department of Surgery, The Ohio State University College of Medicine, Columbus, OH, United States; ^4^ Department of Veterinary Biosciences, The Ohio State University College of Veterinary Medicine, Columbus, OH, United States

**Keywords:** cancer, technologies, diagnostics, therapeutics, novel modalities

This Research Topic “*Novel Modalities in Cancer Diagnostics and Therapeutics*” is the second issue following our previous topic “*New Technologies in Cancer Diagnostics and Therapeutics*” and continuously focuses on the newly emerging areas of cancer diagnostics, drug development, and molecular signaling pathways (Li et al., 2021). Our aim was to collect the latest findings in both basic research and clinic application to contribute to the future direction of drug development or novel therapy. To this end, we received 35 manuscripts and finally 17 articles were selected in this Research Topic.

This Research Topic comprises two review papers, two case reporters and thirteen research articles. Tumor microenvironment (TME) composed of tumor cells, immune cells such as macrophages, and plays an important function in the cancer development, progression, and prognosis. The review article by Liu et al. summarized the functions of tumor-associated macrophages (TAMs) in thyroid cancer and discussed the mechanisms by which TAMs maintain the stemness of thyroid cancer and potential strategies for targeting TAMs to treat thyroid cancer. Another review article by Li et al. highlighted the regulatory mechanisms underlying the crosstalk between *H. pylori* infection and immune cells in TME of gastric cancer. *H. pylori* is well known to induce immune response and inflammation in the stomach and involved in gastric cancer progression. The research demonstrated that the underlying molecular mechanism concerning the crosstalk between *H. pylori* and the TME of Gastric cancer may contribute to develop effective therapy against *H. pylori* -induced gastric cancer. The research article by Wang et al. provided evidence that Interleukin-7 receptor (IL7R) may function as a potential prognostic factor for lung adenocarcinoma and found that IL7R expression was positively correlated with the overall survival and progression-free survival of lung adenocarcinoma patients. Their findings suggest that IL7R inhibits tumor growth by regulating the proportion of immune infiltrating cells in TME. The case report article by Sun et al. presented a case study of a middle-aged male patient with advanced lung adenocarcinoma who underwent a descending surgery after nine cycles of individualized chemotherapy combined with targeted immunotherapy and continued adjuvant chemotherapy. Following a comprehensive analysis, they developed a precise individualized chemotherapy plan and the patient achieved complete response. Wang et al. reported a combined treatment study using anlotinib (a small molecule multi-target tyrosine kinase inhibitor) and celecoxib (COX-2 inhibitor) in a desmoid tumors (DTs) patient with aggressive fibromatosis in the abdominal cavity. They provided evidence that the patient achieved a partial response with mild toxicity and disappeared pain symptoms following a combined therapy.

Hepatocellular carcinoma (HCC) is one of the most common malignant tumors and the leading cause of cancer-related mortality worldwide. The immunotherapy which targets immune checkpoints, such as anti-programmed cell death 1(PD1) targeted immunotherapy, resulted in promising and encouraging effects in the treatment or various tumors. The studies from Wei et al. found that the combination of the FAK inhibitor (VS4718) and an anti-PD1 antibody could suppress tumor progression in murine model for hepatocellular carcinoma (HCC). The combination of the FAK inhibitor VS4718 and anti-PD1 could be a potential therapy for HCC by improving the immune environment, reducing liver fibrosis and simultaneously preventing PD1 from binding to the increased PD-L1 induced by FAK inhibitor. Drug-eluting beads-transarterial chemoembolization (DEB-TACE) has been widely used in unresectable and advanced hepatocellular carcinoma (HCC). Bi et al. assessed that the preliminary outcomes of DEB-TACE loaded with oxaliplatin for the treatment of patients with unresectable or recurrent HCC. The data suggested that DEB-TACE loaded with oxaliplatin-eluting CalliSpheres microspheres could be a safe, feasible, and efficacious palliative regimen in unresectable or recurrent HCC patients. Liu et al. investigated the expression of Consortin, a marker of poor differentiation of blood cells in the peripheral blood of acute myeloid leukemia (AML) patients by analyzing data from public databases and showed that CNST expression was inversely correlated with overall survival among AML patients and the genes negatively correlated with CNST are involved in various immune-related pathways. Mixed lineage leukemia gene rearrangements (MLLr) were found in about 10% of all the AML cases and in 22% of all the acute lymphoblastic leukemia (ALL) cases. Yao et al. investigated the Histone deacetylases inhibitor I1 (HDAC-I1), a chromatin-remodeling agent and found that a marked anti-proliferative effect on MLLr-AML and MLLr-ALL cells. HDAC-I1 inhibited HDAC and activated the hematopoietic cell lineage signaling, suggesting that HDAC-I1 could be a potential epigenetic agent for the treatment of Acute leukemia. Liu et al. developed the hemicyanine-based fluorescent probe ZWZ-3, which can be selectively enriched in the mitochondria of melanoma cells, thus promoting mitochondrial oxidative phosphorylation and inducing apoptosis and autophagy. Their data suggested that ZWZ-3 represents a potential therapeutic agent for detecting and treating melanoma. Qin et al. investigated the properties of polysaccharides of A. bracteate, named ABP and show that ABP increase anti-inflammatory cytokine IL-10, inhibited secretion of pro-inflammatory cytokines (IL-6, IFN-γ, and TNF-α), mitigated oxidative stress, and found that the CAC mice treated with ABP showed smaller tumor size and lower tumor incidence than untreated ones. Using database from TCGA and GEO, Wang et al. identified six risk biomarkers associated with prognosis of oral squamous cell carcinoma (OSCC). The expression of these risk genes in clinical specimens was higher in the non-survival patients than in the well-survival patients, suggesting that those gene signature and nomogram might provide a potential prognostic prediction and therapeutic for OSCC. Zhou et al. reported that miR-320a expression is lower in cisplatin (DDP) resistant cervical cancer (CC), and engineered miR-320a exosomes can attenuate DDP resistance *via* inhibiting Myeloid Cell Leukemia Sequence 1 (MCL1), a pro-survival and pro-proliferative factor which plays a critical role in tumorigenesis. The engineered miR-320a exosomes may represent a new therapy for chemoresistance cervical cancer treatment. Wang et al. studied the impact of sclerostin on malignant progression of Uveal melanoma (UM) and demonstrated that Sclerostin silencing through transfecting specific siRNA could heighten the proliferation, migration, and invasion as well as angiogenesis of human UM cells *via* activating Wnt/β-catenin signaling. Natural products with low toxicity are always important source for drug development. Zhou et al. investigated vanillin-derivate intervention by *in vitro* co-culturing with colorectal cancer cells and exploring the possible underlying mechanism. Their results showed that both vanillin derivatives were effective for *F. nucleatum*-infected Colorectal cancer (CRC) cells by inhibiting proliferation and migration through the E-cadherin/β-catenin pathway, suggesting potential natural product drug candidates for microbe-targeted strategies for the treatment of CRC.

Histone acetylation modification is one of the major significant epigenetic modifications, which are involved in various cellular biological processes including carcinogenesis (Langst and Manelyte, 2015). Dr. Xiangzhi Li’s group has long been committed to studying the function of MOF (males absent on the first), a histone acetyltransferase in a variety of physiological and pathological processes including cancers (Li et al., 2009; Li et al., 2010; Li et al., 2012; Guo et al., 2020; Wang et al., 2021). In this Research Topic, Li’s group further provides exciting evidence for the potential role of MOF in breast cancer (BC) (Zhang et al.) and renal cell carcinoma (RCC) (Guo et al.). Although the abnormal gene expression of MOF has been found in several types of cancers, more studies are still needed to comprehensively understand the function and complex regulatory mechanism of MOF in the initiation and progression of cancers and their relationship to cancer prognosis. Screening and discovering drugs or small molecules that can normalize the intracellular acetylation levels in cancer cells could be a prospective guide for further research and clinical applications.

In conclusion, this Research Topic “*Novel Modalities in Cancer Diagnostics and Therapeutics*” highlights multiple studies for developing potential targets or novel therapeutics for cancer diagnosis and treatment.
